# Strength in Recovery: A Practice-Informed Commentary on Peer Recovery Specialists Facilitating Gambling Recovery Groups

**DOI:** 10.1007/s10899-026-10502-6

**Published:** 2026-04-30

**Authors:** Waganesh Zeleke, Carolyn Hawley, Shruti Sampath, Amy Armstrong

**Affiliations:** Department of Rehabilitation Counseling, College of Health Professions, Virginia Commonwealth University, Richmond, VA USA

**Keywords:** Peer recovery specialists, Gambling recovery, Peer workforce, Trauma-informed care, Strengths-based practice, Commentary

## Abstract

Gambling-related harm is increasingly recognized as a public health concern with significant psychological, relational, and social consequences, yet limited scholarship has examined the integration of Peer Recovery Specialists (PRS) within gambling recovery systems. While peer roles are well established in substance use and mental health contexts, gambling-specific peer integration remains emergent and under-theorized. This manuscript offers a practice-informed commentary on the role of Peer Recovery Specialists facilitating gambling recovery groups within a statewide system of care. Drawing on structured reflective conversations with the full cohort of certified Peer Recovery Specialists (N = 5) embedded within a statewide gambling recovery initiative, the commentary synthesizes practitioner perspectives and implementation lessons. The work is intentionally positioned as descriptive and reflexive rather than evaluative and transparently acknowledges the authors’ embedded roles within the system of care. Peer Recovery Specialists described their work as fundamentally relational, strengths-based, and trauma-informed. Key areas of emphasis included the use of lived experience to foster connection and hope, structured yet flexible facilitation practices, intentional language to reduce stigma and promote psychological safety, and the importance of organizational supports such as supervision, training, and peer networks. By positioning these insights as practice-informed rather than outcome-focused, this commentary offers guidance for organizations seeking to integrate Peer Recovery Specialists into gambling recovery systems and contributes to emerging discourse on gambling-specific peer roles and workforce development.

## Introduction

Gambling-related harm is increasingly recognized as a public health concern with significant psychological, relational, and social consequences (Wheaton et al., 2024). Beyond financial loss, individuals affected by gambling harm often experience stigma, isolation, emotional distress, and disruption to family and community relationships. As access to gambling expands through legalization and digital platforms, recovery-oriented systems of care are increasingly called upon to extend beyond clinical treatment alone and address long-term recovery, connection, and meaning (Penfold & Ogden, 2022; Magidson et al., 2022).

Peer-based recovery supports have become an integral component of contemporary behavioral health systems. Peer Recovery Specialists (PRS)—individuals with lived recovery experience who are trained, certified, and embedded within formal systems of care—offer relational engagement, mutual understanding, and pathways to connection that are often difficult to replicate through traditional services alone (Eddie et al., 2019; Stack et al., 2022). Across substance use and mental health contexts, peer support has been associated with engagement, belonging, hope, and stigma reduction (Ortiz et al., 2021).

Despite this growth, gambling-specific integration of PRS remains comparatively underexamined in both research and practice literature. Gambling recovery presents distinct challenges related to stigma, disclosure of lived experience, role clarity, and workforce development infrastructure. While existing scholarship has documented challenges of peer work, including emotional labor, role ambiguity, and burnout (Moran et al., 2013; Anvari et al., 2022), less attention has been paid to strengths-based facilitation practices and organizational conditions that support sustainability, particularly within gambling recovery systems.

This manuscript responds to this gap by offering a practice-informed commentary on the role of Peer Recovery Specialists facilitating gambling recovery groups within a statewide system of care. Rather than evaluating outcomes or effectiveness, the commentary centers practitioner reflection to describe how PRS understand their professional identities, approach group facilitation, and experience organizational supports. This positioning aligns with calls for transparency, reflexivity, and contextual clarity in peer workforce scholarship, particularly when authors are embedded within employing organizations (Bell et al., 2025).

## Practice Context and Reflective Approach

This commentary is situated within the Virginia Partnership for Gaming & Health (VPGH), a statewide public health initiative housed at Virginia Commonwealth University. VPGH collaborates with state agencies, treatment providers, and community organizations to address gambling-related harm through prevention, treatment, and recovery-oriented supports. A central component of this initiative is the integration of certified Peer Recovery Specialists who facilitate structured gambling recovery groups and collaborate with interdisciplinary partners across the system of care.

The authors are embedded within this initiative and have been directly involved in program development, workforce training, supervision, and reflective learning processes. This insider positionality is acknowledged transparently and informs the reflexive stance of the manuscript, consistent with ethical guidance for peer workforce scholarship (Bell et al., 2025).

The insights presented draw from facilitated reflective conversations conducted with the full cohort of Peer Recovery Specialists (*N* = 5) who were actively facilitating gambling recovery groups during the implementation period. These conversations occurred as part of routine professional learning and quality improvement activities. Two facilitated group reflection sessions were held via videoconference, each lasting approximately two hours, with prompts focused on professional identity, facilitation practices, relational dynamics, and organizational supports perceived as meaningful or sustaining.

An appreciative, strengths-based orientation guided reflection, emphasizing effective practices and conditions that support sustainability rather than deficit-focused evaluation (Cooperrider, 1986; Cooperrider et al., 2008). Notes and transcripts were reviewed collaboratively by the author team to identify recurring areas of emphasis that surfaced across reflections. While informed by qualitative sense-making approaches (Braun & Clarke, 2006), this process was used to organize practice insights rather than to generate analytic findings.

Institutional review processes determined that these activities constituted quality improvement and program reflection rather than human subjects research. Participation was voluntary, and Peer Recovery Specialists were informed that synthesized reflections might be shared in scholarly venues.

## Practice-Informed Insights from Peer Recovery Specialists

### Professional Identity and Relational Purpose

Peer Recovery Specialists consistently described their professional identities as deeply intertwined with lived recovery experience, resilience, and relational purpose. Recovery was understood not as a completed achievement but as an ongoing process that informed empathy, humility, and boundary awareness. One PRS reflected, *“The resilience I’ve gained from my lived experience allows me to bring hope and strength to others*,*”* emphasizing how personal recovery served as a foundation for relational engagement rather than authority.

Connection and relationship were described as central to PRS work. Participants emphasized that many individuals affected by gambling harm enter recovery spaces carrying shame and isolation, and that peer facilitation offers a unique opportunity to foster trust and belonging. As one PRS shared, *“I value my ability to connect with people and help others feel hopeful in an otherwise seemingly hopeless situation.”* Another reflected, *“My story became my most powerful tool—not because it was about me*,* but because it helped others feel less alone.”* These reflections align with recovery-oriented frameworks that conceptualize healing as relational and meaning-centered (Penfold & Ogden, 2023).

### Strengths-Based and Trauma-Informed Facilitation Practices

PRS described their facilitation practices as intentionally strengths-based and trauma-informed. Storytelling emerged as a commonly referenced practice, used selectively to normalize experiences, reduce shame, and model recovery. Importantly, participants emphasized that storytelling was not about centering themselves. One PRS explained, *“I make sure the group is not about me—I’m just facilitating the conversation.”*

Facilitation was described as **structured yet flexible**, balancing predictability with responsiveness to participant needs. PRS emphasized that structure helped create emotional safety while preserving autonomy. As one participant noted, *“We don’t tell people what they must do—we let them guide their own pathway.”* This approach aligns with trauma-informed principles that emphasize choice, collaboration, and empowerment (SAMHSA, 2014; Najavits et al., 2023).

Language use was identified as a subtle but powerful element of practice. PRS intentionally reframed conversations away from deficit-based narratives toward contextual understanding. One participant shared, *“I try not to focus on what’s wrong with them*,* but on what happened.”* Such language was perceived as reducing shame and fostering psychological safety, consistent with trauma-informed and stigma-reduction frameworks (Hart et al., 2024; Kleinman et al., 2024).

### Organizational Supports and Sustainability

Organizational supports were consistently identified as essential to PRS effectiveness and well-being. Participants emphasized the value of ongoing training, peer networking, and structured supervision. Group supervision and peer consultation spaces were described as critical for reflection, emotional processing, and skill development. One PRS noted, *“Being surrounded by other peers really helps sustain me in this work.”*

Workplace culture emerged as a key factor in sustainability. PRS described environments characterized by flexibility, mutual support, and recognition of lived experience as protective against burnout. Several participants noted that facilitating groups was often personally uplifting. As one shared, *“I found myself being uplifted in the very group I facilitated.”* These reflections echo broader literature emphasizing the importance of institutional strategies that support peer specialists as both professionals and individuals in recovery (Moran et al., 2013; Anvari et al., 2022).

### Implications for Gambling Recovery Systems

The reflections presented suggest several implications for integrating Peer Recovery Specialists into gambling recovery systems. First, intentional role design is critical. Effective peer integration requires clarity regarding scope of practice, boundaries, training, and supervision, extending beyond the inclusion of lived experience alone (Bell et al., 2025).

Second, training models should incorporate group facilitation skills, trauma-informed practice, reflective supervision, and narrative approaches to recovery. PRS emphasized ongoing professional development as central to confidence, ethical practice, and sustainability.

Finally, organizational infrastructure and workplace culture are foundational. Access to supervision, peer networks, and flexible policies supports workforce retention and well-being. At a policy level, gambling recovery systems would benefit from recognizing PRS as integral members of interdisciplinary teams and supporting sustainable funding, certification pathways, and advancement opportunities.

### Reflexive Limitations and Conclusion

This manuscript is intentionally positioned as a practice-informed commentary rather than an evaluation. The insights presented reflect practitioner experience within a specific organizational context and do not include service user perspectives or outcome data. The appreciative orientation may have foregrounded strengths while limiting exploration of systemic tensions.

Nevertheless, by transparently positioning these insights as practice-based and contextually grounded, this commentary contributes to emerging discourse on gambling-specific peer integration. As gambling recovery services continue to evolve, ethically grounded and well-supported peer roles represent a promising and necessary component of comprehensive systems of care.
